# The impact of infrared beak treatment on turkey tom and hen beak length and performance to 12 weeks of age

**DOI:** 10.1016/j.psj.2022.101737

**Published:** 2022-01-19

**Authors:** S. Struthers, T. Fiss, H.L. Classen, S. Gomis, E. Herwig, K. Schwean-Lardner

**Affiliations:** ⁎ The Roslin Institute and Royal (Dick) School of Veterinary Studies, University of Edinburgh, Edinburgh EH25 9RG, UK; † Department of Agriculture, Horticulture, and Engineering Science, Scotland's Rural College, Edinburgh EH25 9RG, UK; ‡ Department and Animal and Poultry Science, University of Saskatchewan, Saskatoon, Canada S7N 5A8; § Department of Veterinary Pathology, Western College of Veterinary Medicine, University of Saskatchewan, Saskatoon, Canada S7N 5B4

**Keywords:** beak shape, body weight, feed intake, injurious pecking, poult

## Abstract

Controlling injurious pecking in commercial turkeys remains a significant challenge to producers and the industry. Infrared beak treatment is an effective method of controlling injurious pecking in chickens; however, the effects of infrared beak treatment on turkey performance are still largely unknown. Two experiments were conducted to determine the impact of infrared beak treatment on the beak length and performance of turkeys raised to 12 wk of age. Experiment 1 tested both toms (n = 236) and hens (n = 324), while Experiment 2 focused on hens (n = 608). Poults for each experiment were assigned to 1 of 2 beak treatments: infrared beak treated (**IR**) on the day of hatch at a commercial hatchery or sham untreated control (**C**). Data collected included beak length, body weight, feed intake, feed efficiency, and mortality. Data were analyzed using a 1 or 2-way ANOVA, followed by Tukey's range test for mean separation when interactions were found. Results showed that beak length (Experiment 1 only) was significantly shorter in IR poults from 2 to 12 wk of age. In the same experiment, IR toms had lighter body weight than C toms, but IR hens were heavier than C hens from 2 to 4 wk of age. By 12 wk, IR poults were heavier than C poults, regardless of gender. In experiment 2, IR hens had lighter body weight from 2 to 4 wk of age. In conclusion, infrared beak treatment had minimal effects on feed intake, feed efficiency, or mortality over the 12-wk periods of both experiments.

## INTRODUCTION

Beak treatment is one of the most effective management practices used in commercial poultry production to help control cannibalism and feather pecking. The practice is continually criticized from an animal welfare perspective as some beak treatment methods have been associated with acute pain (Gentle, 1991), reduced growth (Gentle et al., 1997), reduced expression of beak-related behaviors (Marchant-Forde et al., 2008), and the formation of neuromas (which may cause chronic pain; Breward and Gentle, 1985). However, the reductions in growth and behavior are often minor and short-lived and must be considered in relation to the positive impacts of beak treatment, such as increased livability and reduced cannibalism (Guesdon et al., 2006; Struthers et al., 2019a).

One method of beak treatment used in commercial turkey production is infrared beak treatment. The process involves placing day-of-hatch birds into a head-holding fixture and exposing their beak tips to a noncontact, high-intensity infrared light (Glatz, 2005). The infrared light penetrates the rhamphotheca (external keratin layer) and damages the keratin-producing cells in the epidermis, thereby inhibiting further growth of the beak tissue (Glatz, 2005). Unlike more traditional methods of beak treatment, infrared beak treatment does not result in the immediate loss of the beak tissue, but rather, the treated portion of the beak erodes and sloughs off one to 2 wk post-treatment, allowing the bird time to adapt to the change in beak shape.

When turkeys are beak treated using electric or hot-blade trimming, the effects on performance are variable. Denbow et al. (1984) reported that body weight did not differ between treated toms (electric trimming at 1 d of age) and control toms, but feed intake was higher in treated birds, while Cunningham et al. (1992) reported that electric beak trimming at 1 d of age resulted in higher body weight and feed intake in turkey toms but not in hens. Leighton et al. (1985) found that turkey hens that were electrically trimmed at 1 d of age had reduced body weight at older but not younger ages. Studies have yet to be reported on with respect to infrared beak treatment in turkeys; however, some previous research on layer pullets report a reduction in body weight and feed intake in infrared beak treated pullets (Gentle and McKeegan, 2007; Marchant-Forde et al., 2008). These reductions typically coincide with the time that the beak tissue is sloughing and are no longer apparent 4 wk post-treatment. More recent studies have found that infrared beak treatment had minimal impacts on pullet body weight, feed intake, and feed efficiency during early life (Struthers et al., 2019a).

Research that has been conducted on infrared beak treatment thus far has been collected from egg-production pullets and hens, and there is an absence of scientific studies examining the effects of infrared beak treatment on turkeys. Therefore, the objective of this project was to determine the effect of infrared beak treatment on the beak length and production performance of turkey tom and hens raised to 12 wk of age. To the author's knowledge, this is the first study reported investigating infrared beak treatment in turkeys and its effect on performance.

## MATERIALS AND METHODS

The protocols for these experiments were approved by the University of Saskatchewan Animal Research Ethics Board and all birds were cared for as specified in the Guide to the Care and Use of Experimental Animals by the Canadian Council of Animal Care (2009). The impact of infrared beak treatment on turkey tom and hen performance was evaluated in 2 experiments.

### Birds and Housing

#### Experiment 1

Five hundred sixty Nicholas Select turkeys (236 toms and 324 hens; Aviagen, WV) were sexed at a commercial hatchery. All poults were then randomly allocated to one of 2 beak treatment groups: infrared beak treated (**IR**) or sham untreated control (**C**). Infrared beak treatment was applied immediately post-hatch using the Poultry Service Processor (Nova-Tech Engineering LLC, Willmar, MN) at lamp power 35 with no mirror (which resulted in only the top beak being treated). Control poults were placed onto the same equipment as treated birds, but their beaks were not exposed to the infrared light. The poults were then transported to the research facility and randomly allocated to 16 pens (4 replicate pens per treatment). Poults were housed at a density of 32 kg/m^2^ based on their predicted 12-wk body weight, resulting in 27 toms or 38 hens per pen (Aviagen, 2015).

Pens measured 3 m × 3 m, and an 8-m circumference brooder ring was used for the first 10 days. Each pen was provided with a supplemental feeder and drinker for the first week and a heat lamp for the first 2 wk. During the brooding period, wood shavings were used as bedding, and after brooding, wheat straw was provided to the pens equally when litter quality was poor. Age-appropriate commercial crumble diets were provided ad libitum in a tube feeder (1 per pen; 36 cm diameter until 4 wk and 44 cm thereafter). Water was provided ad libitum using a 38-cm bell drinker for the first 4 wk and 56 cm after. The temperature was set at 30°C for the initial brooding period (first week) then reduced by approximately 2°C per week to reach a final temperature of 13°C at 9 wk of age. Humidifiers were used for the first 2 wk to maintain a range of 50 to 75% RH. The light was provided using incandescent bulbs. For the first 5 d, the photoperiod was 23L:1D (40 lux). Starting at 6 d of age, the photoperiod was reduced by 1 h per day to reach a final photoperiod of 18L:6D (5 lux) at 10 d of age. This photoperiod was maintained for the remainder of the experiment. Light intensity was also reduced daily by 6 lux starting at 4 d of age.

#### Experiment 2

This experiment used only Nicholas Select turkey hens (n = 608). Beak treatments, husbandry, housing, and environmental conditions were identical to those used in Experiment 1. Poults were randomly allocated to 16 floor pens resulting in 8 replicate pens per treatment.

### Data Collection

During Experiment 1, the extent of sloughing was recorded from every bird in 2 replicates (76 hens and 54 toms per treatment) by visual examination from 3 d of age until sloughing was complete. Poults were individually examined, and their top beaks were identified as either 1) intact with no visible signs of sloughing, 2) partial sloughing where the epidermis had partially sloughed off, or 3) complete sloughing where the entire beak tip had sloughed off.

Beak length was also determined during Experiment 1 for all birds in 2 replicates per treatment (76 hens and 54 toms) at 0, 1, 2, 4, 8, and 12 wk of age. For weeks 0, 1, 2, and 4, photographs of the beak were taken using the Nova-Tech beak scale (Nova-Tech Engineering LLC) and a Canon PowerShot SD1200IS Camera (Canon Canada Inc., Mississauga, ON, Canada). Photographs were analyzed to calculate beak length (distance between the anterior end of the nares to the end of the upper beak) and overall growth using ImageJ analysis software (version 1.52, National Institutes of Health, MD). At 8 and 12 wk of age, beak photographs were taken using an Olympus OM-D E-M10 camera (Olympus Imaging America Inc., Centre Valley, PA) and beak length was determined using a scale also present in the photo.

Body weight and feed intake for both experiments were determined on a pen basis at 0, 1, 2, 3, 4, 8, and 12 wk of age. Mortality was recorded daily, and all dead or culled birds were sent to an independent diagnostic laboratory for necropsy. Turkeys from both experiments that showed minor signs of pecking (either aggressive or feather) had pine tar applied to the wounded area to discourage further pecking, while turkeys that underwent severe pecking were removed from the trial and placed in a recovery pen or euthanized at the discretion of the animal technicians. From these data, average body weight, average feed intake, feed efficiency (feed to gain ratio) with and without mortality correction, and percent mortality were calculated.

### Statistical Analyses

Experiment 1 was designed as a 2 × 2 factorial arrangement of beak treatment and gender, in a completely randomized design, with 4 replicates per treatment. Experiment 2 was a one-way analysis of variance with 8 replicates per beak treatment in a completely randomized design. Due to an insufficient number of replicates for statistical analysis, beak length data is presented as observations only. Data were analyzed using PROC MIXED (SAS 9.4, Cary, NC) with Tukey's range test to separate means. Percentage data were checked for normality using PROC UNIVARIATE (SAS 9.4, Cary, NC) and log-transformed (data log + 1) when necessary. Differences were considered significant when *P* ≤ 0.05 and a trend was noted when 0.05 < *P* ≤ 0.10.

## RESULTS

### Beak Characteristics

#### Beak Sloughing

**Figure 1:**
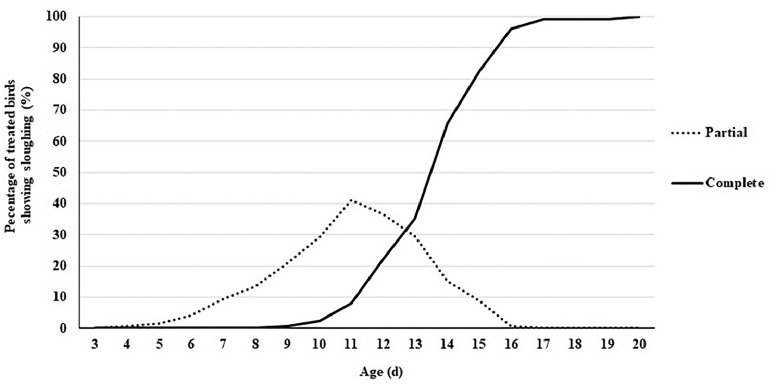
Percentage of infrared beak treated turkeys showing partial or complete sloughing from 3 to 20 d of age.

**Figure 2:**
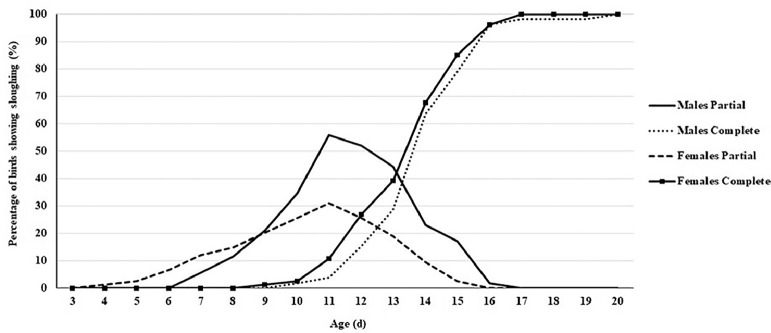
Percentage of infrared beak treated male and female turkeys showing partial or complete sloughing from 3 to 20 d of age.

During the first experiment, sloughing off the treated beak tissue was first seen at 4 d of age, with 0.80% of poults showing partial sloughing (Figure 1). Completely sloughed beaks were first observed at 9 d of age, with 0.80% of poults showing complete sloughing. All beaks completed sloughing by 20 d of age. Female poults began to slough sooner than males and were faster at sloughing throughout (Figure 2). Sloughing was first noted in hens at 4 d of age and toms at 7 d. Sloughing was complete in hens by 17 d of age and 20 d for toms.

#### Beak Length and Growth

Infrared beak treatment reduced beak length from 2 to 12 wk of age in the first experiment (Table 1). IR turkeys also had less overall beak growth throughout the 12-wk period than C turkeys (11.50 vs. 22.25 mm, respectively).

**Table 1:** Effect of beak treatment and gender^1^ on beak length (mm) and growth (mm) of Experiment 1 turkeys from 0 to 12 wk of age.

Age (wk)	Beak treatment			Gender			SEM
	IR	C	*P*-value	Male	Female	*P*-value	
Beak length, mm							
0	6.14	6.11	-	6.10	6.16	-	0.019
1	7.32^b^	7.94^a^	-	7.74	7.52	-	0.134
2	5.76^b^	8.18^a^	<0.01	7.19	6.75	-	0.473
4	8.94^b^	11.03^a^	<0.01	10.05	9.92	-	0.414
8	15.78^b^	21.67^a^	<0.01	19.60	17.84	-	1.245
12	17.64^b^	28.36^a^	<0.01	25.72^a^	20.28^b^	<0.01	2.311
Beak growth, mm							
0–12	11.50^b^	22.25^a^	<0.01	19.62	14.62	-	2.319

Abbreviations: C, sham untreated control; IR, infrared beak treated.

a,b Means within a main effect with different superscripts are significantly different (*P* ≤ 0.05).

1 Data is presented as observations only.

### Production Data

#### Body Weight

Infrared beak treatment had minor effects on the body weight of Experiment 1 turkeys, with a significant difference noted only at 12 wk of age (Table 2). At this age, IR turkeys were heavier than C turkeys (10.48 vs. 10.16 kg, respectively). C hens were heavier during the second experiment than IR hens at 2, 3, and 4 wk of age. This trend continued with body weight at 8 wk of age (*P* = 0.07), and no differences after that. Interactions between beak treatment and gender in Experiment 1 were noted at 2 and 4 wk of age (Table 3). At 2 wk of age, C toms were heavier than IR and C hens. At 4 wk, toms were heavier than hens, regardless of beak treatment.

**Table 2:** Effect of beak treatment and gender on the production performance of Experiment 1 and 2 turkeys from 0 to 12 wk of age.

Age (wk)	Beak treatment			Gender			Interaction	SEM
	IR	C	*P*-value	Male	Female	*P*-value	*P*-value	
Body weight (kg), Experiment 1								
0	0.06	0.06	0.90	0.06	0.06	0.06	0.85	0.000
1	0.16	0.16	0.33	0.17^a^	0.16^b^	0.01	0.06	0.003
2	0.39	0.39	0.63	0.41^a^	0.38^b^	<0.01	0.01	0.006
3	0.72	0.70	0.44	0.74^a^	0.68^b^	0.05	0.95	0.015
4	1.24	1.25	0.65	1.34^a^	1.15^b^	<0.01	0.01	0.025
8	5.15	5.08	0.29	5.58^a^	4.65^b^	<0.01	0.15	0.125
12	10.48^a^	10.16^b^	0.01	11.58^a^	9.06^b^	<0.01	0.21	0.332
Body weight (kg), Experiment 2								
0	0.06	0.06	0.70	-	-	-	-	0.001
1	0.14	0.14	0.16	-	-	-	-	0.002
2	0.33^b^	0.34^a^	0.03	-	-	-	-	0.002
3	0.63^b^	0.64^a^	<0.01	-	-	-	-	0.004
4	1.09^b^	1.12^a^	0.01	-	-	-	-	0.006
8	4.48	4.55	0.07	-	-	-	-	0.017
12	8.35	8.38	0.74	-	-	-	-	0.042
Feed intake (kg/bird), Experiment 1								
0–1	0.11	0.11	0.77	0.11^a^	0.10^b^	0.01	0.01	0.002
1–2	0.28	0.28	0.83	0.29^a^	0.27^b^	<0.01	0.37	0.004
2–3	0.45	0.44	0.66	0.47^a^	0.42^b^	0.03	0.93	0.009
3–4	0.73	0.74	0.24	0.79^a^	0.67^b^	<0.01	0.01	0.017
4–8	6.63	6.51	0.36	7.16^a^	5.98^b^	<0.01	0.33	0.164
8–12	12.50	12.73	0.63	13.84^a^	11.38^b^	<0.01	0.79	0.382
Feed intake (kg/bird), Experiment 2								
0 – 1	0.09	0.09	0.24	-	-	-	-	0.001
1–2	0.26^b^	0.27^a^	<0.01	-	-	-	-	0.002
2–3	0.46	0.45	0.06	-	-	-	-	0.003
3–4	0.71^b^	0.73^a^	0.03	-	-	-	-	0.004
4–8	5.96	6.09	0.09	-	-	-	-	0.039
8–12	10.68	10.81	0.29	-	-	-	-	0.059
Feed to gain (mortality corrected), Experiment 1								
0–12	1.98	2.03	0.23	1.95^b^	2.07^a^	<0.01	0.74	0.025
Feed to gain (mortality corrected), Experiment 2								
0 – 12	2.17	2.18	0.49	-	-	-	-	0.007
Mortality, Experiment 1								
Total mortality	7.26	9.89	0.27	9.26	7.89	0.56	0.27	1.140
Mortality from pecking	1.91	6.10	0.09	3.70	4.28	0.57	0.99	1.150
Pecking deterrent	8.23^b^	26.17^b^	<0.01	12.04	22.36	0.06	0.69	3.650
Mortality, Experiment 2								
Total mortality	4.28	6.91	0.37	-	-	-	-	1.419
Mortality from pecking	1.64	4.60	0.16	-	-	-	-	1.025
Pecking deterrent	2.63	3.95	0.64	-	-	-	-	1.171

Abbreviations: C, sham untreated control; IR, infrared beak treated.

a,b Means within a main effect with different superscripts are significantly different (*P* ≤ 0.05).

**Table 3:** Interaction between beak treatment and gender for body weight (kg) of Experiment 1 turkeys.

Age (wk)	Male IR	Male C	Female IR	Female C
2	0.40^ab^	0.42^a^	0.39^bc^	0.36^c^
4	1.31^a^	1.36^a^	1.17^b^	1.14^b^

Abbreviations: C, sham untreated control; IR, infrared beak treated.

a,b,c Means within a row with different superscripts are significantly different (*P* ≤ 0.05).

#### Feed Intake and Efficiency

Beak treating turkeys using infrared beak treatment did not alter the amount of feed consumed over the 12-wk period of Experiment 1 (Table 2). Experiment 2 IR hens had lower feed intake from 1 to 2 and 3 to 4 wk. In the same experiment, there was a trend for IR hens to have lower feed intake from 2 to 3 (*P* = 0.06) and 4 to 8 wk (*P* = 0.09) compared to C hens. There were no differences in feed efficiency between IR and C turkeys during either experiment. Interactions between beak treatment and gender in Experiment 1 were noted from 0 to 1 and 3 to 4 wk of age (Table 4).

**Table 4:** Interaction between beak treatment and gender for feed intake (kg/bird) of Experiment 1 turkeys.

Age (wk)	Male IR	Male C	Female IR	Female C
0–1	0.10^ab^	0.12^a^	0.11^ab^	0.10^b^
3–4	0.77^a^	0.82^a^	0.68^b^	0.66^b^

Abbreviations: C, sham untreated control; IR, infrared beak treated.

a,b Means within a row with different superscripts are significantly different (*P* ≤ 0.05).

#### Mortality

For both experiments, no differences in total mortality were found between IR and C turkeys over the 12-wk periods (Table 2). However, infrared beak treatment tended to reduce mortality due to pecking during Experiment 1 (*P* = 0.09). Infrared beak treatment also significantly reduced the percentage of live birds that needed to have a pecking deterrent applied due to injurious pecking and aggression in Experiment 1 (8.2 vs. 26.1 %, for IR and C turkeys, respectively). There were no differences in total mortality between toms and hens during Experiment 1; however, there was a tendency for a greater percentage of hens to require the application of a pecking deterrent compared to toms (*P* = 0.06).

## DISCUSSION

Beak treatment is performed in many commercial poultry species in an effort to reduce the physical damage and mortality that can result from cannibalism and injurious pecking. By removing the sharp, hook-shaped beak tip, birds are less effective at grasping the feathers and tissues of other birds (Gentle et al., 1997; Dennis and Cheng, 2012). The beak length data from the present study suggests that the infrared beak treatment setting used was effective at shortening beak length as the birds aged. This is similar to Marchant-Forde et al. (2008), who found significant differences in beak length between infrared beak treated and control pullets as soon as 1-wk post-treatment. Beak growth over the 12-wk period was found to be reduced in IR turkeys in the present study. Inhibiting beak regrowth is important as it decreases the potential that birds will require a second beak trim later in life and it ensures that the beak tip does not grow back enough that birds re-gain their ability to damage the skin and plumage of other birds (Marchant-Forde and Cheng, 2010).

When there is a reduction in feed intake following beak treatment, there is often a corresponding reduction in body weight (Marchant-Forde et al., 2008; Marchant-Forde and Cheng, 2010). Reductions in both parameters not only have economic consequences but can also compromise welfare if beak treated birds are not able to grasp successfully and consume feed or are in pain because of the shortened and blunted beak shape (Gentle et al., 1982; Prescott and Bonser, 2004; Marchant-Forde et al., 2008). In the first experiment of the present study, no differences in body weight or feed intake were observed between the IR and C turkeys. However, in the second experiment, IR hens weighed and ate significantly less than the C hens from 2 to 4 wk of age. Previous literature has also observed differences in pullet body weight and feed intake during the 2- to 4-wk period following infrared beak treatment (Gentle and McKeegan, 2007; Marchant-Forde et al., 2008; Marchant-Forde and Cheng, 2010; Struthers et al., 2019a). During this time, the treated beak tissue was sloughing and the reduction in body weight and feed intake observed in Experiment 2 may have been due to altered feeding behavior as the IR hens adapted to the change in beak shape. It is unlikely that the reductions in feed intake and, therefore, body weight were due to the birds being in pain as there were no differences in pecking force (an indicator of pain) between treated and untreated turkeys during this period (Fiss et al., 2018).

Looking beyond the first 4 wk of life, previous studies conducted with laying hens and broiler breeders have suggested that infrared beak treatment does not affect final body weight (Gentle and McKeegan, 2007; Marchant-Forde et al., 2008; Struthers et al., 2019a). In the present study, Experiment 1 IR turkeys had heavier body weights than C turkeys at 12 wk of age. However, in the second experiment, no differences in body weight were observed at the same age.

Larger experiments conducted with laying hens have observed significant reductions in mortality when hens are beak treated (Guesdon et al., 2006; Mertens et al., 2009; Riber and Hinrichsen, 2017). Although no statistical differences in overall mortality and mortality due to pecking were observed between beak treatments over the 12-wk period, the mortality from the 2 experiments agrees with previous studies (Riber and Hinrichsen, 2017; Struthers et al., 2019b) and helps justify that infrared beak treatment reduces the ability to grasp and damage the tissues and plumage. This is especially important as the bird's age and become more at risk for pecking injuries. It is also possible that differences in mortality may have been confounded by the application of a pecking deterrent when birds were bullied. It has been found that when a bitter-tasting substance was applied to the plumage of laying hens, severe feather pecking was decreased and that birds learned to avoid the feathers of conspecifics (Harlander-Matauschek et al., 2009).

In conclusion, the results of this study suggest that although infrared beak treated turkeys had lower body weight and feed intake up to 4 wk post-treatment, infrared beak treatment did not have any long-term impacts on the productivity of turkey toms and hens. It is clear that the treated turkeys were able to adapt to the change in beak shape once sloughing was complete. Infrared beak treatment simultaneously improved welfare by reducing mortality due to pecking.
